# Excitatory Amino Acid Transporters in Physiology and Disorders of the Central Nervous System

**DOI:** 10.3390/ijms20225671

**Published:** 2019-11-12

**Authors:** Anna R. Malik, Thomas E. Willnow

**Affiliations:** 1 Nencki Institute of Experimental Biology, 02-093 Warsaw, Poland; 2 Max-Delbrueck-Center for Molecular Medicine, 13125 Berlin, Germany; willnow@mdc-berlin.de

**Keywords:** glutamate, glutathione, oxidative stress, intracellular trafficking, neurodegeneration, excitotoxicity, EAAT, GLAST, GLT-1, EAAC1

## Abstract

Excitatory amino acid transporters (EAATs) encompass a class of five transporters with distinct expression in neurons and glia of the central nervous system (CNS). EAATs are mainly recognized for their role in uptake of the amino acid glutamate, the major excitatory neurotransmitter. EAATs-mediated clearance of glutamate released by neurons is vital to maintain proper glutamatergic signalling and to prevent toxic accumulation of this amino acid in the extracellular space. In addition, some EAATs also act as chloride channels or mediate the uptake of cysteine, required to produce the reactive oxygen speciesscavenger glutathione. Given their central role in glutamate homeostasis in the brain, as well as their additional activities, it comes as no surprise that EAAT dysfunctions have been implicated in numerous acute or chronic diseases of the CNS, including ischemic stroke and epilepsy, cerebellar ataxias, amyotrophic lateral sclerosis, Alzheimer’s disease and Huntington’s disease. Here we review the studies in cellular and animal models, as well as in humans that highlight the roles of EAATs in the pathogenesis of these devastating disorders. We also discuss the mechanisms regulating EAATs expression and intracellular trafficking and new exciting possibilities to modulate EAATs and to provide neuroprotection in course of pathologies affecting the CNS.

## 1. Introduction

**Figure 1:**
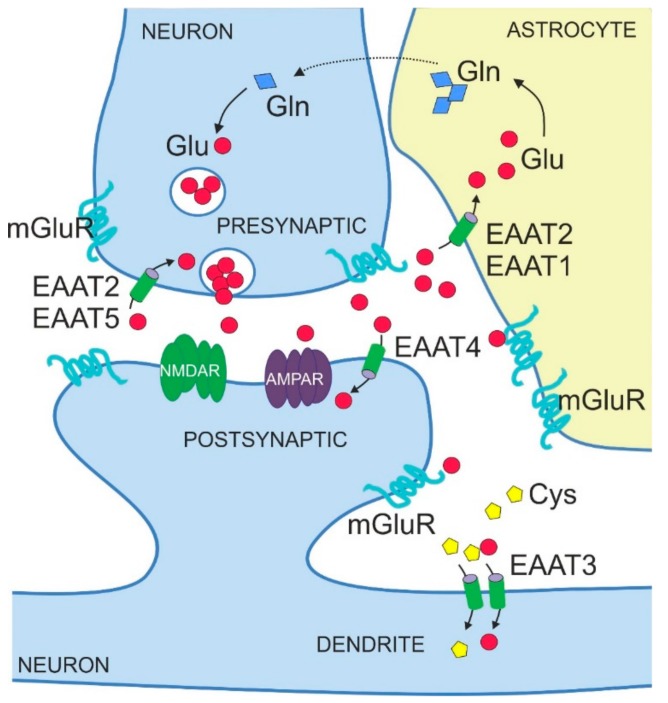
Expression and activities of Excitatory amino acid transporters (EAATs) in neurons and astrocytes. Glutamate (Glu; red dots) is released by neurons at the presynaptic side of the synapse and acts on neuronal and glial glutamate receptors (α-amino-3-hydroxy-5-methyl-4-isoxazolepropionic acid receptors, AMPARs; *N*-methyl-d-aspartate receptors, NMDARs; metabotropic glutamate receptors, mGluRs). Released glutamate may also diffuse out of the synaptic cleft causing activation of distant receptors. Extracellular glutamate is removed by EAATs present in astrocytes and neurons. In astrocytes, glutamate is converted into glutamine (Gln, blue squares), which is shuttled back to neurons by the activity of neutral amino acid transporters. In addition to glutamate, EAAT3 also transports the glutathione precursor cysteine (Cys; yellow symbol) into neurons, required to produce glutathione. This figure is a schematic depiction of neuronal and astrocytic expression patterns for EAATs. In vivo, EAAT1 and EAAT4 are only present in synapses in the cerebellum while EAAT5 is restricted to neurons in the retina.

The amino acid glutamate is the major excitatory neurotransmitter in the central nervous system (CNS). It is required for essential brain functions, such as learning and memory [1,2]. Neurons release glutamate at the presynaptic side of the synapse to transmit signals to other neurons that bind released glutamate via specialized receptors at the postsynaptic side, that is, metabotropic glutamate receptors (mGluRs) acting via G proteins and ionotropic glutamate receptors acting as glutamate-gated ion channels (Figure 1). Ionotropic glutamate receptors include kainate receptors, *N*-methyl-d-aspartate receptors (NMDARs) and α-amino-3-hydroxy-5-methyl-4-isoxazolepropionic acid receptors (AMPARs). Released glutamate can also bind to and stimulate glutamate receptors on non-neuronal cell types in the brain, in particular on astrocytes (Figure 1). Binding of glutamate to its receptors elicits molecular events that lead to ion flux and/or activation of signalling cascades, thereby transmitting the signal into the receiving cell.

Excessive accumulation of extracellular glutamate causes neuronal cell death. Therefore, maintaining glutamate levels within a physiological range, that is, 10 µM in the cerebrospinal fluid (CSF) and up to 30 µM in the extracellular space [3], is vital. Also, proper signal transmission requires keeping glutamate in the synaptic cleft at defined concentrations (<20 nM [3]). Since glutamate released by neurons is generally not metabolized in the extracellular space, the maintenance of normal glutamatergic neurotransmission and the prevention of glutamate-induced toxicity depends on active glutamate uptake into glial cells and neurons.

It had been known since 1970s that a high affinity glutamate uptake system existed in the mammalian brain [4]. Further investigations led to the identification of excitatory amino acids transporters or EAATs. EAATs belong to the solute carrier 1 (SLC1) family of transmembrane amino acid transporters and transport glutamate and aspartate across the plasma membrane [5,6,7,8,9]. Five different EAATs have been identified (Table 1), designated EAAT1 [5], EAAT2 [9], EAAT3 [6], EAAT4 [7] and EAAT5 [8]. Multiple nomenclatures are used with the rodent homologues called GLAST (glutamate-aspartate transporter, corresponding to the human EAAT1), GLT-1 (glutamate transporter 1, EAAT2) and EAAC1 (excitatory amino acid carrier 1, first cloned from rabbit, EAAT3). Genes names follow the SLC terminology and are SLC1A3 for EAAT1, SLC1A2 for EAAT2, SLC1A1 for EAAT3, SLC1A6 for EAAT4 and SLC1A7 for EAAT5. For simplicity, we will use the terms EAAT1-5 in this review.

## 2. Molecular Properties of EAATs

**Figure 2:**
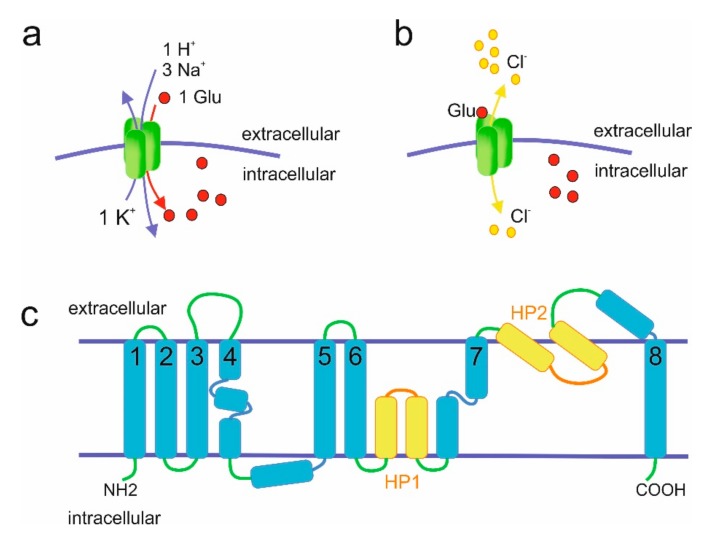
Molecular properties of EAATs. (**a**) Stoichiometry of glutamate transport by EAATs. As exemplified for glutamate (Glu), amino acid uptake is driven by co-transport of three sodium ions and one proton and by counter-transport of one potassium ion. (**b**) Chloride conductance through EAATs. EAATs act as chloride-permeable ion channels activated by binding glutamate. (**c**) Transmembrane topology of glutamate transporters based on the structure of bacterial GltPh transporter. The presumed topology consists of 8 transmembrane domains (blue symbols 1 to 8) and two hairpin loops (HP, yellow symbols). Scheme based on Reference [16].

The molecular properties of mammalian EAATs have been extensively discussed elsewhere [10,11,12] and will only be briefly summarized here. EAATs-mediated glutamate transport is sodium-dependent and coupled with H^+^ and K^+^ transport (Figure 2). Amino acid uptake is driven by the co-transport of three sodium ions and one proton, as well as the counter-transport of one potassium ion [13,14]. In addition to transporting amino acids, EAATs also act as glutamate-gated chloride channels [15].

Mammalian EAATs differ in transport rates and substrate affinities [11]. Despite the fact that results are affected by expression systems used (e.g., *Xenopus* oocytes versus HEK cells), EAAT3 seems to be the most effective glutamate transporter with a turnover rate of 90 s^−1^ [17], followed by EAAT1 (16 s^−1^) [18] and EAAT2 (14.6 s^−1^) [19]. EAAT4 and EAAT5 exhibit very low turnover rates, which were calculated as <3 s^−1^ for EAAT4 [20] and even too low to be determined for EAAT5 [21]. Since EAAT4 exhibits very low transport rate yet the highest affinity for glutamate, a role for this protein in modulation of diffusion rather than in uptake of glutamate was proposed [20]. With low transport rates and affinity EAAT5 does not seem to play major role in glutamate transport either [8,21]. Instead, it may function as a glutamate-gated chloride channel [7]. In fact, EAAT4 and EAAT5 have the largest chloride conductance amongst the EAATs [20,21] and may act as glutamate-gated chloride channels rather than glutamate transporters [22,23,24].

EAATs are multi-pass transmembrane proteins (Figure 2). Transmembrane topologies of EAATs were first proposed based on cysteine substitutions experiments for rat EAAT2 [25] and human EAAT1 [26] (reviewed in References [27,28]) and it is generally accepted that the structures of EAATs are similar to the structure of glutamate transporter ortholog from *Pyrococcus horikoshii*, GltPh, with 8 transmembrane domains and 2 hairpin loops (Figure 2) [16] that was solved in 2004. The crystal structure of mammalian EAAT1 solved in 2017 indeed confirmed the structural similarity to GltPh [29]. EAATs seem to naturally occur as trimeric molecules [30], although pentameric structures were also reported [31]. EAATs complexes are usually homomultimers but formation of EAAT3 and EAAT4 heterotrimers was also observed in recombinant overexpression systems [32]. Although the trimer represents the mature, functional form present at the plasma membrane [30,33], the three subunits in the complex function independently [34,35,36].

## 3. Expression Patterns of EAATs

The five mammalian EAATs show distinct localization patterns, both in terms of expression in different brain regions and cell types but also in terms of subcellular distribution (Table 1 and Figure 1). EAAT1 and EAAT2 are mainly expressed in astrocytes [37,38,39], whereas EAAT3, EAAT4 and EAAT5 are predominantly found in neurons [22,40,41]. EAAT1 and EAAT2 are also detected in oligodendrocytes [42,43]. With respect to expression domains in the mature mammalian brain, EAAT2 and EAAT3 are abundant throughout various brain structures such as hippocampus, cortex and striatum [39,40]. By contrast, EAAT1 and EAAT4 are mostly expressed in the cerebellum, in an astrocyte-lineage Bergmann glia and in neuronal Purkinje cells, respectively [44,45,46,47]. EAAT1 is additionally expressed by retinal glial cells, called Müller cells [48]. The expression of EAAT5 is limited to retina [8], in particular to photoreceptors and bipolar cells [41].

EAATs also show subtype-specific subcellular localizations (Figure 1, Table 1). Thus, EAAT1 and 2 display a prominent perisynaptic localization with distinct punctate distribution [37,49], a pattern in line with their relevance for synaptic functions. Of note, immunostaining for EAAT1 and 2 is also seen at the endfeet of astrocytes on blood vessels [37,49]. Some studies also reported the presence of otherwise glial EAAT2 in neurons, specifically in presynaptic axon terminals [50,51]. Amongst the neuronal EAATs, EAAT5 is localized pre-synaptically [52], whereas EAAT3 and EAAT4 are localized post-synaptically with EAAT3 present predominantly in cell soma and dendrites [40] and EAAT4 in dendrites and dendritic spines [46,47].

The expression levels of EAATs show dynamic changes during development. In particular, levels of astrocytic EAAT1 and EAAT2 increase from birth to adulthood [53,54,55]. Glutamate uptake by astrocytic transporters to maintain low glutamate levels in the synaptic cleft represents an important functional element of glutamatergic synapses (Figure 1) [56,57,58]. Thus, it is not surprising that upregulation of EAAT1 and EAAT2 in astrocytes parallels the development of neuronal networks, synaptogenesis and initiating glutamatergic transmission as the primary excitatory system. Although an increase in expression during brain development is observed for both astrocytic EAATs, mature astrocytes of the forebrain express mainly EAAT2 [44]. By contrast, EAAT1 is predominantly expressed earlier in development by immature astrocytes and radial glia [59,60] but restricted to the cerebellum in the adult brain [44].

Among the neuronal EAATs, EAAT4 [61] and EAAT5 [41] increase in expression during development. The increase in EAAT4 levels corresponds to dendritic arborization of Purkinje cells, a process associated with development of the molecular layer of the cerebellum [61,62]. The dynamics of EAAT5 expression during ontogeny corresponds to the time course of onset of activity of photoreceptors [41].

Taken together, the expression levels of EAAT1, 2, 4 and 5 increase as brain circuits develop and mature. At the same time, only modest changes in the expression of EAAT3 occur with age [40]. Thus, in contrast to the other EAATs, the expression and presumably also function of EAAT3 does not appear to be directly linked to neuronal activity and synapse formation.

## 4. Physiological Functions of EAATs

EAATs were originally identified as transporters for excitatory amino acids glutamate and aspartate, with glutamate being the physiologically most relevant substrate for uptake into the cells of the mammalian brain. Accordingly, a role for EAATs in removing glutamate from the extracellular space to prevent aberrant excitability and neurodegeneration was proposed. Glutamate taken up into astrocytes is converted to physiologically inert glutamine and shuttled back to excitatory neurons, a process known as the glutamate-glutamine cycle (Figure 1). Early mouse studies documented the importance of glial transporters EAAT1 and 2 for glutamate clearance in the brain under basal conditions. Thus, silencing the expression of EAAT1 and 2 by chronic administration of antisense oligonucleotides elevated extracellular glutamate levels in the murine brain and caused neurodegeneration characteristic of excitotoxicity [63]. Later studies on gene-targeted (KO) mouse models confirmed that EAAT2 is crucial for bulk glutamate uptake from the extracellular space and for maintaining its physiological levels to prevent excitotoxicity. EAAT2-KO mice showed selective neuronal degeneration in the hippocampal CA1 region, spontaneous epileptic seizures and premature death, phenotypes that can be explained by an excess of glutamate in the extracellular space [64].

Gene inactivation of EAAT1 had less profound effect on brain morphology and function. In detail, EAAT1-deficient mice showed mild motor discoordination but normal cerebellar architecture and synaptic transmission [65]. Further studies linked loss of EAAT1 in mice to locomotor hyperactivity in a novel environment, to abnormal sociability and to impaired learning, behaviour interpreted as ‘schizophrenia-related’ abnormalities [66,67] and a recent report showed that loss of EAAT1 alters the firing patterns of a subpopulation of cerebellar Purkinje cells and causes discrete neuronal cell loss [68].

Mice deficient in neuronal EAATs show relatively mild phenotypes under basal conditions, which questions the relevance of those transporters for bulk glutamate removal from extracellular space in the healthy brain. In detail, silencing EAAT3 in the brain by chronic administration of antisense oligonucleotides did not increase extracellular glutamate levels or cause overt neurodegeneration [63]. Also, EAAT3 knockout mice did not develop obvious neurodegeneration in the cerebral cortex, hippocampus or cerebellum. Still, they showed a mild behavioural phenotype and were more prone to die in an experimental model of epilepsy [69]. Still, EAAT3 may play a role in preventing glutamate spillover from the synapse and in fine-tuning synaptic transmission [70,71]. Moreover, since glutamic acid serves as a precursor for synthesis of γ-aminobutyric acid (GABA) in inhibitory interneurons that express EAAT3 [72], EAAT3 activity may be crucial for GABA production. Indeed, knocking down EAAT3 in the brain decreased GABA levels [73] and caused staring–freezing episodes and epileptiform brain activity as shown by video-EEG. Interestingly, EAAT3 has an additional transport activity involved in neuronal stress responses. In contrast to other EAATs, EAAT3 is capable of transporting cysteine into neurons [74,75]. EAAT3-mediated uptake of cysteine provides the rate-limiting substrate for neuronal synthesis of glutathione (GSH) [76,77], a reactive oxygen species scavenger that protects cells from oxidative stress. Cysteine transport emerges as a major physiological function of EAAT3, as loss of this transporter results in a reduction in neuronal glutathione levels and in increased susceptibility to oxidative stress [78]. Moreover, EAAT3-knockout mice show age-dependent loss of dopaminergic neurons in the substantia nigra, a defect that coincides with increased oxidative stress and inflammation in these animals [79]. Another study also showed decreased glutathione levels and increased oxidative stress in the cortex and hippocampus, as well as cognitive impairment in EAAT3-depleted mice [80].

As for possible functions of EAAT4 in neurons of the cerebellum, studies in knockout mice suggest that it has a specialized role in clearing glutamate released at climbing fibres’ synapses [81] and that depletion of EAAT4 leads to alterations in the firing patterns of Purkinje cells [68]. For EAAT5, no knockout animals have been reported thus far. Still, an important role in regulating synaptic transmission in the retina was attributed to EAAT5 which acts as a presynaptic glutamate receptor mediating chloride conductance [23,52].

In summary, a role in maintaining brain homeostasis by removal of excess glutamate from the extracellular space can clearly be assigned to glial EAAT2, while the activities of other four EAATs seem to be more specialized and locally restricted. Thus, the actions of the latter transporters are linked to fine-tuning synaptic transmission in the respective brain structures where they are expressed. Additionally, EAAT3 plays a role in protecting neurons from oxidative stress.

## 5. Regulation of Expression Levels of EAATs

The activities of EAATs are dynamically regulated. Regulation is mainly achieved through the control of expression levels and of subcellular localization. The molecular mechanisms in control of EAATs genes transcription have extensively been discussed in a recent review [82]. Here, we will focus on external cues that induce EAATs gene expression and on cellular signalling pathways that transduce such signals. In general, factors influencing expression levels of EAATs include growth factors, hormones, neuronal transmission and various stress factors. Moreover, a blinded screen of 1040 FDA-approved drugs and nutritionals revealed that many β-lactam antibiotics are potent stimulators of EAAT2 gene expression [83]. One of those agents, ceftriaxone, was later used in several studies as a means to upregulate EAAT2 levels [84]. Little is known about the regulation of expression levels of EAAT4 and EAAT5. Therefore, the subsequent paragraphs will mostly discuss expression regulation of EAAT1-3.

### 5.1. Regulation by Growth Factors, Polypeptides and Hormones

Early studies showed that neurons release factors that increase protein levels of EAAT1 and EAAT2 in cultured glia [85,86,87]. One of the first neuron-secreted factors shown to induce transcript and protein levels of EAAT1 and 2 in cultured glia was pituitary adenylate cyclase-activating polypeptide (PACAP) [87]. Growth factors that induce expression of EAAT1 and EAAT2 are transforming growth factor alpha (TGFα) and epidermal growth factor (EGF) [88,89,90,91] that were shown to increase both mRNA and protein levels of the transporters. Additional growth factors studied for their potential to induce EAAT1 and EAAT2 levels gave conflicting results. For example, platelet-derived growth factor (PDGF) increased EAAT2 transcript and protein in some studies in primary astrocytes cultures [89] but this effect was not observed in astrocytic preparations of high purity [88]. The effects of glial cell-derived neurotrophic factor (GDNF) and brain-derived neurotrophic factor (BDNF) are unclear as well. While neither GDNF nor BDNF affected EAAT1 and EAAT2 expression in one study [89], GDNF increased protein levels of EAAT1 and 2 [92] while BDNF raised EAAT2 protein levels [93] in others. Finally, basic fibroblast growth factor (bFGF, FGF-2) was shown, (i) to increase transcript and protein levels of EAAT1 [90]; (ii) to induce mRNA and protein levels of both EAAT1 and 2 but only upon simultaneous inhibition of ERK signalling [89]; or (iii) to have no influence on EAAT1 or 2 levels [88]. Interestingly, an in vivo study showed that FGF-2 restored EAAT1 and EAAT2 protein levels that were decreased in posttraumatic stress disorder [94]. This finding indicates that stimulatory effects of some growth factors on EAAT1 and EAAT2 expression strongly depend on the pathophysiological context in the brain.

Among hormones, oestrogen and selective oestrogen receptor modulators tamoxifen and raloxifene induce EAAT1 and EAAT2 transcript and protein levels [95,96,97]. The mechanisms underlying this induction are elusive and may affect release of growth factors, such as TGFα [98] and/or transactivation of EGF receptor, as shown for G protein-coupled oestrogen receptor GPR30 activation [99]. Also, levels of EAAT3 transcript and protein were induced by oestrogen in C6 cells [100], through a mechanism that again seemed to be indirect and to involve induction of FGF-2. Other hormones implicated in regulation of EAATs levels are glucocorticoids which increase EAAT2 mRNA and protein levels in cultured cortical astrocytes [101] and in the rat hippocampi [102].

Several signal cascades upregulate EAAT1 and EAAT2. Firstly, the cAMP-protein kinase A (PKA)-CREB signalling pathway is involved in transcriptional regulation of EAAT1 and 2. Thus, PACAP-driven increase of EAAT1 levels requires PKA activity [87]. Also the stimulatory effect of TGFα on EAAT1 levels is mediated through PKA [89]. Moreover, activation of the EAAT2 gene promoter by application of tamoxifen or TGFα is blocked by a PKA inhibitor [97]. In line with these findings, cAMP analogues directly induce EAAT1 and 2 transcript and protein in glial cell cultures [85,87,88,89]. Treatment of cultured astrocytes with the cAMP inducer forskolin also increases protein levels of EAAT1 and EAAT2 [101]. Finally, a stimulatory effect of CREB overexpression on EAAT2 gene promoter activity [97] and direct binding of CREB to the EAAT2 gene promoter [99] have been documented.

Calcium signalling engaging protein kinase C (PKC) also regulates EAATs expression, particularly of EAAT2. PKC inhibition abolishes the stimulatory effect of PACAP, TGFα and PDGF on EAAT2 protein levels but has no effect on EAAT1 increase driven by growth factors and PACAP [87,89]. Finally, activation of extracellular signal-regulated kinases (ERK) and Phosphoinositide 3-kinase (PI3K)-Akt signal transduction pathways plays a crucial role in inducing EAAT2 gene expression. For example, the effects of TGFα and PDGF on EAAT2 levels are blocked by Akt inhibition [89]. Also, BDNF-triggered increase of EAAT2 levels is blocked by inhibiting ERK [93]. Moreover, the stimulatory effects of oestrogen receptors agonists and modulators on EAAT2 levels are abolished when PI3K or ERK are inhibited [95,99].

Various pathways inducing EAAT1 and EAAT2 gene expression converge on the transcription factor NF-κB. Inhibiting NF-κB completely abolished the stimulatory effects of PACAP, TGFα and EGF on EAAT1 gene expression [89,103] as well as the induction of EAAT2 by PACAP, TGFα, EGF and PDGF [89,104]. Along this line, EGF triggers binding of NF-κB to the EAAT2 gene promoter [104]. Overexpression of NF-κB p65 increases EAAT1 gene promoter activity, as well as the levels of EAAT1 transcript and protein [103]. Similar effects were observed for EAAT2 with overexpression of NF-κB p65 increasing EAAT2 gene promoter activity as well as EAAT2 protein levels [105]. Also β-lactam ceftriaxone used as a means to induce EAAT2 gene expression exerts its action via NF-κB [84]. It is worth mentioning that some factors decrease the levels of EAATs. Thus, TNFα decreased EAAT1 levels in cultured primary astrocytes [106] and endothelins inhibited transcription of EAAT2 gene [107].

Putative regulation of EAAT3, 4 and 5 levels by growth factors is elusive. EAAT3 seems to be mostly regulated at the level of intracellular trafficking rather than expression (see Section 6). Still, EAAT3 protein levels are increased by TNFα in neuronal cultures [108] and by EGF-like trophic factor Neuregulin 1 in cultured neurons and in vivo [109].

### 5.2. Regulation by Neuronal Activity

Functionally, EAATs are part of the glutamatergic system in the brain. Thus, it is reasonable that their expression is regulated by excitatory neurotransmission. This fact is very well documented for glial EAATs that are active components of a glutamatergic synapse (Figure 1). Early studies showed that the protein levels of EAAT1 and EAAT2 decreased after pharmacological blockade of neuronal activity in neuronal-glial co-cultures [86]. Moreover, glutamate up-regulated EAAT2 transcripts in cultured astrocytes [110] and both EAAT1 and EAAT2 transcripts in rat optic nerves [111]. EAAT2 gene promoter activity and protein levels in co-culture systems depended on direct contact between astrocytes and neuronal axons [112]. These observations were confirmed in vivo, as glutamatergic denervation, induced by cortical lesion, caused the reduction of EAAT1 and EAAT2 protein levels in rats [113]. Also, degeneration of axons decreased the activity of the EAAT2 gene promoter and the amounts of EAAT2 protein [112]. Moreover, whisker stimulation, a paradigm driving neuronal activation in the barrel cortex of mice, caused a transient increase in EAAT1 and EAAT2 protein levels in this brain region, a finding again pointing to a direct relationship between neuronal activity and protein levels of EAAT1 and 2 [114]. Of note, such a link was not observed for EAAT3 [114].

How do neuronal activity and glutamatergic signalling induce EAATs gene expression? Some clues come from pharmacological studies using glutamate receptors antagonists and agonists. In detail, blocking both ionotropic and metabotropic glutamate receptors reduced protein levels of EAAT2 [112], suggesting direct involvement of signalling pathways triggered by glutamate in EAATs expression control. Additional studies focusing on metabotropic glutamate receptors showed that activation of mGluR2/3 positively modulated the protein levels of EAAT1 and 2 [91,115]. In agreement with these findings, transcript and protein levels of EAAT1 and EAAT2 protein levels were reduced in mGluR3-KO mice [116]. Similarly to the induction of EAATs by growth factors, EAAT2 induction by mGluR2/3 agonist requires the activity of ERK and PI3K pathways [91]. The same fact was shown for increase of EAAT1 protein levels triggered by agonists of mGluR2/3 receptors under hypoxic conditions, which was mediated through PI3K, ERK and NF-κB [115].

EAAT3 mRNA levels are decreased in mice deficient for mGluR2 [116] but no further information is available concerning direct effects of glutamatergic signalling on its expression levels. Regarding EAAT4 and 5 expression levels, we are not aware of published work showing their expression regulation by glutamatergic signalling.

### 5.3. Regulation by Hypoxia and Oxidative Stress

Hypoxic conditions have been studied extensively for their potential to regulate glutamate transporters, as this putative regulation could have important consequences for brain pathologies. Perhaps not surprisingly, the results varied depending on the cell type and hypoxia paradigm used. Also, the results varied between in vitro and in vivo setups, with the latter allowing the capture of the effects of a complex interplay between various cell types exposed to ischemic conditions in the brain. Thus, EAAT2 gene transcription was induced by hypoxia in the cultured GT1-7 cell line [117]. By contrast, both EAAT1 and 2 were downregulated at the levels of mRNA and protein following hypoxia in cultured astrocytes [118]. Short oxygen-glucose deprivation (OGD) increased protein levels of EAAT2 but not EAAT1 [108] in mixed glia-neuronal cortical cultures. Finally, brief OGD induced EAAT2 protein levels in cultured astrocytes [119] but failed to stimulate glutamate uptake, suggesting no influence of hypoxia on glial glutamate transporter activity [108].

In organotypic hippocampal slices subjected to an in vitro model of ischemia-reperfusion, transcript levels for EAAT1 initially dropped to be induced at later time points, while EAAT2 gene transcription was downregulated [120]. The latter observation is in line with the fact that global brain ischemia decreases EAAT2 protein levels [121]. Also, chronic perinatal hypoxia reduces EAAT1 and EAAT2 protein levels but this effect is only seen during early postnatal development [122].

Contrary to EAAT1 and 2, robust induction of EAAT3 by hypoxia is obvious. EAAT3 is induced at the transcript level by hypoxia in cultured C6 cells [117] and at the protein level by short OGD in mixed glial-neuronal and in pure neuronal cultures [108]. Of note, in contrast to observations from astrocytic cultures, brief OGD also promotes EAAT3 activity as it increases glutamate uptake into cultured neurons [108]. In an in vitro model of ischemia-reperfusion, transcript levels for EAAT3 were transiently up-regulated during the reperfusion phase [120]. In vivo, ischemia-reperfusion lead to oxidative stress and a concomitant transient increase in EAAT3 immunoreactivity in the hippocampus [123].

As oxidative stress is a direct result of hypoxia, it is tempting to speculate that the upregulation of EAAT3 expression in hypoxia or under ischemia-reperfusion conditions is triggered by oxidative stress. In agreement with this hypothesis, evolutionarily conserved antioxidant responsive elements (AREs) are found in the promoter of the EAAT3 encoding gene [124]. AREs play a crucial role in preventing oxidative damage as they are involved in inducing expression of various protective genes under oxidative stress. Nuclear factor (erythroid-derived 2)-like 2 (Nrf2) is one of the transcription factors binding to AREs. Activators of Nrf2 or exogenous expression of Nrf2 increase EAAT3 gene transcription in C6 cells [124]. Along these lines, selective expression of Nrf2 in neurons in vivo raises levels of both EAAT3 and glutathione in mice [124], further supporting the concept that the induction of the EAAT3 gene is part of the oxidative stress response. Oxidative stress is a hallmark of various brain pathologies and largely contributes to the clinical outcome of CNS disorders, such as ischemic stroke, epilepsy and neurodegenerative diseases. Accordingly, a potential protective role for EAAT3 in these conditions can be envisioned (see Section 7).

## 6. Regulation of Subcellular Localization of EAATs

Since EAATs exert their actions at the cell surface, dynamic regulation of intracellular trafficking and targeting to the plasma membrane emerges as one of the main mechanisms controlling EAATs activities. Moreover, glutamate transporters, especially EAAT2, localize to cholesterol-rich lipid raft microdomains, which is crucial for their activity [125]. More recent work now showed that in addition to intracellular trafficking between plasma membrane and endocytic compartment, EAAT2 is also regulated by lateral diffusion that is crucial for shaping synaptic transmission [126]. In brief, EAAT2 molecules are highly mobile at the surface of astrocytes, with their mobility being affected by neuronal and glial activities. Interestingly, impairing EAAT2 mobility influenced excitatory postsynaptic currents [126].

Jointly, the presence within lipid rafts, surface diffusion, and, foremost, intracellular trafficking all seem to be important processes influencing the activity of EAATs. These mechanisms were especially well studied for EAAT2 and EAAT3 and for the latter appear to be the main route of regulating transporter activity. The most potent factors to influence subcellular localization of EAATs are glutamate, trophic factors and PKC activators. Treatment with the PKC activator phorbol 12-myristate 13-acetate (PMA) causes internalization of EAAT2 in cultured C6 cells, a process dependent on a unique carboxyl-terminal sequence not present in EAAT3 [127]. Also in astrocytes, addition of PMA elicits an immediate internalization of EAAT2, the occurrence of cytoplasmic ‘clusters,’ corresponding depletion of EAAT2 in the plasma membrane and a decrease in glutamate uptake [128]. Internalization of EAAT2 is prevented by expression of a dominant-negative form of dynamin, suggesting that PMA treatment is coupled to clathrin-dependent endocytosis of EAAT2 [128]. A similar effect as with PMA is observed when treating astrocytes with glutamate. Upon glutamate treatment, EAAT2 is internalized and forms intracellular ‘clusters,’ identified as accumulations of EAAT2 in early and recycling endosomes [129]. As of now, the molecular mechanisms underlying sorting of EAAT2 remain largely unknown. Moreover, as glutamate positively regulates EAAT2 gene transcription, the overall outcome of glutamate release on functional expression of this transporter in vivo is difficult to predict. Also, our knowledge about targeting this transporter or other EAATs to lipid rafts and regulating surface diffusion is very limited. Still, it was shown that overexpression of ciliary neurotrophic factor (CNTF) in the brain triggered glycosylation and re-distribution of EAAT1 and 2 to the lipid raft fraction. Translocation to rafts was accompanied by an increased clearance of glutamate from the extracellular space [130]. Finally, EAAT2 lateral diffusion in the astrocytic plasma membrane was stimulated by neuronal activity and by treatment with glutamate [131].

**Figure 3:**
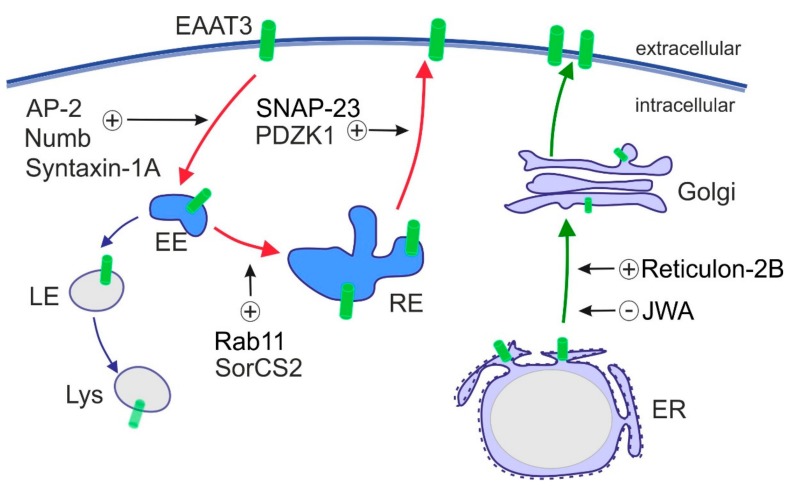
Intracellular trafficking of EAAT3. Newly synthesized EAAT3 moves via the biosynthetic pathway from the endoplasmic reticulum (ER) through the Golgi compartments to the plasma membrane (exocytic route, green arrows). From the cell surface, EAAT3 is constitutively endocytosed and directed through early endosomes (EE) and recycling endosomes (RE) back to the plasma membrane (recycling route, red arrows). Alternatively, internalized EAAT3 may be directed from EE to late endosomes (LE) and further to lysosomes (Lys) for proteolytic degradation (blue arrows). Proteins involved in promoting (+) or inhibiting (−) EAAT3 trafficking are indicated at the respective translocation steps.

In contrast to other EAATs, intracellular trafficking of EAAT3 plays a major role in controlling EAAT3 activity and is well studied. Plasma membrane exposure of EAAT3 decreased after kainic acid stimulation [132,133] and increased upon activation of PDGF pathway, PI3K and PKC [134,135,136,137]. PKC activation is presumably the most potent modulator of EAAT3 trafficking that promotes EAAT3 targeting to the plasma membrane. EAAT3 travels via the exocytic route through endoplasmic reticulum (ER) and Golgi and through the recycling route, the latter being crucial for dynamic regulation of cell surface levels of EAAT3 (Figure 3). Proteins that influence exocytic trafficking of EAAT3 are Reticulon-2B and JWA, which regulate the ER exit of EAAT3 [138,139]. The recycling route involves clathrin-dependent endocytosis from the cell surface, followed by recycling through the Rab11+ compartment back to the plasma membrane [140]. Proteins involved in endosomal trafficking and regulation of cell surface expression of EAAT3 include SNARE proteins SNAP-23 [141] and Syntaxin-1A [133], small GTPase Rab11 [140], clathrin adaptor Numb [142], scaffold protein PDZK1 and clathrin adaptor complex AP-2 [143], as well as the specialized sorting receptor SorCS2 that assists EAAT3 in its recycling route [144]. Sorting through Rab11+ recycling endosomes is central for maintaining EAAT3 activity as Rab11 dysfunction slows EAAT3 trafficking to the cell surface and impairs cysteine uptake and glutathione biosynthesis [145]. Along these lines, loss of the intracellular sorting receptor SorCS2 re-directs EAAT3 to late endosomes. This fate causes aberrant proteolytic degradation of EAAT3, decreasing its amount at the neuronal cell surface [144]. Disturbances in these sorting pathways and aberrant subcellular localization of EAAT3 are observed in CNS disorders, such as epilepsy, Huntington’s disease and Parkinson’s disease (see Section 7 and Table 2 for further details).

Not much is known about intracellular trafficking of other EAATs. Anchoring of EAAT4 to the plasma membrane is mediated by GTRAP41 (β-III spectrin) and GTRAP48 (Rho guanine nucleotide exchange factor 11), which stabilize EAAT4 at the cell membrane and increase glutamate uptake [146]. In line with these findings, mutant Δ39 β-III spectrin fails to stabilize EAAT4 at the plasma membrane [147] and mutant L253P β-III spectrin disrupts post-Golgi trafficking of EAAT4, thereby preventing its targeting to the plasma membrane [148]. These molecular mechanisms have crucial implications for the pathogenesis of spectrin-related ataxias (see Section 7.7).

## 7. EAATs in the CNS Pathology

Altered expression and function of EAATs, foremost EAAT1 and EAAT2, have been linked to psychiatric disorders, in particular to schizophrenia. This association is not surprising given the causative role of altered synaptic transmission in psychiatric conditions. The reader is referred to excellent recent reviews on this aspect of EAATs function [149,150,151,152].

Disturbances in glutamate handling and elevations of extracellular glutamate concentrations have also been implicated in multiple CNS disorders that are characterized by excitotoxicity and cell death, such as neurodegenerative diseases, ischemic stroke and epilepsy [153,154,155,156,157,158,159,160,161,162]. In line with that notion, the protective role of EAATs in brain diseases can be often, although not always, explained by preventing increases in extracellular glutamate concentrations. This protective role of EAATs in various CNS disorders will be discussed in detail in the following sections and is briefly summarized in Table 2.

**Table 2:** Main central nervous system (CNS) diseases linked to EAATs.

Disease	EAAT	EAAT’s Activity Crucial for Preventing Pathology/Proposed Pathological Mechanism	Changes in Levels/Distribution in Course of the Disease	References
epilepsy	EAAT2	EAAT2 prevents aberrant excitability and excitotoxic damage by removing excess glutamate	decreased levels	[64,163,164,165,166,167,168,169,170,171,172]
	EAAT3	EAAT3 has a protective role through involvement in GABA synthesis and in protection from oxidative damage	changes in subcellular distribution; increased levels in surviving neurons	[69,73,78,144,164,165,173,174,175,176]
Alzheimer’s disease	EAAT2	EAAT2 prevents excitotoxic damage by removing excess glutamate	decreased levels	[177,178,179,180,181,182,183,184,185,186,187]
Parkinson’s disease	EAAT3	EAAT3 protects neurons from oxidative damage	shift to the plasma membrane in a mouse model of PD	[79,188]
Huntington’s disease	EAAT2	EAAT2 prevents excitotoxic damage by removing excess glutamate	decreased levels	[189,190,191,192,193,194,195,196]
	EAAT3	EAAT3 protects neurons from oxidative damage	decreased levels; aberrant intracellular trafficking	[145,193,196]
multiple sclerosis	EAAT2	EAAT2 may be potentially protective by preventing excitotoxic damage by removing excess glutamate	inconsistent results	[111,197,198,199,200]
amyotrophic lateral sclerosis	EAAT2	EAAT2 prevents excitotoxic damage by removing excess glutamate	decreased levels, aberrant splicing	[83,201,202,203,204,205,206,207,208,209,210,211,212,213,214,215]
episodic ataxia (EA6)	EAAT1	EAAT1 mutants show impaired glutamate uptake and alterations in anion conductance	mutations in EAAT1 coding gene identified in patients	[216,217,218,219,220,221]
spinocerebellar ataxias (SCA1, SCA5)	EAAT4	Reduced EAAT4 activity impairs spontaneous activity of Purkinje cells and causes neuronal death	decreased levels in SCA1; aberrant intracellular trafficking and decreased levels in SCA5	[148,222,223,224,225]
spinocerebellar ataxias (SCA5, SCA7)	EAAT1	EAAT1 prevents excitotoxic damage by removing excess glutamate	decreased levels	[224,225,226,227,228]
ischemic stroke	EAAT2	EAAT2 prevents excitotoxic damage by removing excess glutamate	decreased levels	[229,230,231,232,233]
	EAAT3	EAAT3 protects neurons from oxidative damage	increased levels	[231,234,235,236]

### 7.1. Epilepsy

Epilepsy is a group of neurological disorders characterized by the occurrence of unprovoked seizures [237], by aberrant neuronal excitability and by loss of neurons. Although the causes of epilepsy may be many, glutamate—as the predominant excitatory neurotransmitter in the brain—undoubtedly plays a crucial role in the initiation and spread of seizure activity and in epileptogenesis, a chronic process that leads to the development of epilepsy [153]. Moreover, elevations in extracellular glutamate concentrations, resulting from epileptic seizures, further contribute to excitotoxic damage and cell death. In a microdialysis study in human subjects, increased extracellular glutamate levels were found in the epileptogenic hippocampus just before and during clinical seizures [238]. Interestingly, increased glutamate levels sustained after the seizure, specifically in the epileptogenic hippocampus. This observation suggested possible malfunctioning and/or downregulation of glutamate transporters in the epileptic brain. The importance of glial glutamate uptake for preventing epileptic seizures and neuronal cell death was also directly proven by early studies in mice with targeted disruption of EAAT2 encoding gene [64].

There is ample evidence that epileptic seizures influence the expression levels of EAATs. Conceptually, modulation of expression may be a consequence of enhanced glutamate release and neuronal activity during epilepsy. Moreover, epileptic seizures cause neuronal cell loss and gliosis [163]. Glia activation and proliferation as such might directly influence brain levels of glial glutamate transporters, contributing to the changes in the expression levels of EAAT1 and EAAT2 in epilepsy. On the other hand, neuronal cell death in the course of epilepsy leads to a reduction in total levels of EAAT3 in the affected brain. Thus, multiple factors have the potential to impact the highly dynamic changes in EAATs levels in the course of epilepsy. In most studies, glial EAATs increased shortly after seizure, likely reflecting acute activation of astrocytes but decreased in the chronic phase later on. For example, status epilepticus (SE) triggered by intrahippocampal application of kainic acid significantly increased hippocampal EAAT2 levels one day post-SE. However, at later timepoints, EAAT2 levels dropped to levels even lower than in control brains [163]. In a chronic model of epilepsy, the expression of EAAT1 and EAAT2 in the hippocampus increased within 24 h of pentylenetetrazol (PTZ) kindling but returned to basal levels 30 days after the last seizure [164]. In humans with temporal lobe epilepsy with hippocampal sclerosis, an overall loss of EAAT2 immunoreactivity was observed in the hippocampus when compared with the non-sclerotic brain tissue [165].

In agreement with findings from pharmacological epilepsy models, EAAT2 expression decreases in rat brains after traumatic brain injury (TBI), a condition that also leads to epileptic seizures [166]. Finally, a mouse model of tuberous sclerosis—a genetic disorder associated with epilepsy—shows decreased expression and function of astrocyte glutamate transporters EAAT1 and EAAT2 and an associated increase in extracellular glutamate levels and excitotoxic neuronal cell death [167,168]. Taken together, the levels and activities of glial EAATs are decreased in the chronic phase of epilepsy in experimental rodent models and in patients, a phenomenon that likely contributes to brain damage and epileptogenesis.

The role of EAAT2 in preventing aberrant excitability and epilepsy is especially well documented. Ubiquitous [64] as well as astrocyte-specific EAAT2 gene knockout [169] lead to seizures and mortality in mice. In the converse situation, overexpression of EAAT2 in astrocytes reduces chronic seizure frequency and mortality in a pilocarpine model of epilepsy. Also, neuronal degeneration in hippocampus after status epilepticus is significantly reduced [170]. Furthermore, inducing EAAT2 expression in a model of post-TBI epilepsy by treatment with ceftriaxone ameliorates symptoms [166]. Similarly, ceftriaxone treatment restores the levels of EAAT2 in a mouse model of tuberous sclerosis which is associated with decreased extracellular glutamate levels in the hippocampus and reduced neuronal cell death [171]. When initiated before the onset of seizures, ceftriaxone treatment even prevents development of epileptic phenotype and improves survival in the tuberous sclerosis mouse model [171]. Another strategy to increase EAAT2 levels in vivo is the inhibition of Hsp90, which prevents EAAT2 degradation and suppresses spontaneous recurrent seizures in a mouse model of temporal lobe epilepsy [172]. Based on the above findings in experimental models, inducing expression of EAAT2 emerges as a promising therapeutic approach to preventing development of epilepsy in patients.

Involvement of EAAT3 in the pathogenesis of epilepsy is more elusive. The majority of reports describe increases in EAAT3 transcript or protein levels acutely after seizures but a decrease in chronic epilepsy. In acute epilepsy models, EAAT3 expression increased in rats following lithium-pilocarpine SE [173] and amygdala kindling [174]. These changes were interpreted as an attempt to compensate for the abnormally high extracellular glutamate levels caused by recurrent seizures. In chronic epilepsy models, more dynamic changes in EAAT3 expression levels were observed. In the PTZ kindling model, EAAT3 transiently increased within 24 h of treatment [164] to decrease at later timepoints. Lithium-pilocarpine-induced SE also caused increased EAAT3 immunoreactivity in the hippocampus after 24h, which returned to control levels during the latent period in the hippocampal CA fields but remained increased in the granule cell layer of the dentate gyrus [175]. Of note, in the course of epilepsy, the total levels of neuronal EAAT3 in the brain decrease due to extensive neuronal cell death. Thus, the net decrease of brain EAAT3 levels may reflect the severity of neuronal death and may largely depend on the brain region studied. In line with this assumption, the overall EAAT3 immunoreactivity in human epileptic hippocampi decreased concurrent with neuronal degeneration but the percentage of EAAT3-positive cells increased in the CA2 and granule cell layer, subareas of the hippocampus relatively resistant to epilepsy-induced pathology [165]. These findings were further supported by a recent report showing an increase in EAAT3 immunoreactivity in surviving neurons of the CA2 region in human epileptic hippocampi [144].

In line with the hypothesis that EAAT3 is mainly regulated at the level of intracellular trafficking, rather than expression, changes in its intracellular localization in epilepsy were observed. In the murine PTZ-kindling model, EAAT3 distribution changed with an apparent shift from a small vesicles to the cell membrane fraction [144] reflecting a change in subcellular distribution. Following kainic acid-induced epileptic seizures in rats, neuronal EAAT3 translocated early after injection to form perinuclear deposits [132]. Interestingly, pilocarpine- or kainate-induced seizures increase EAAT3 transcript levels in the dendrites of hippocampal pyramidal cells, suggesting possible regulation of EAAT3 activity by dendritic targeting of transcripts as well [176].

The role of EAAT3 in epileptic seizures seems to be modulatory rather than causative. Support for this hypothesis stems from findings in mice genetically deficient for EAAT3, which lack overt behavioural manifestations of seizures [69,78]. Also, a loss-of-function mutation in the EAAT3 gene causing loss of surface expression and transport activity of EAAT3 does not cause epileptic phenotypes in humans [239]. However, knock down of EAAT3 expression with siRNA causes a mild seizure phenotype in rats [63,73]. Moreover, in the kindling model of epilepsy, easily-kindled rats have lower levels of EAAT3 as compared with epilepsy-resistant animals, suggesting that the convulsive threshold might be determined by EAAT3 levels [164].

Based on the various functions associated with EAAT3, it is conceivable that the actual role of EAAT3 in epilepsy may not involve glutamate removal but rather other activities of this transporter. EAAT3 plays a role in synthesis of GABA [73] and EAAT3 loss decreases the levels of this inhibitory neurotransmitter. As alterations in inhibitory neurotransmission have an obvious impact on the course of epilepsy, this activity provides a possible explanation for the mild epileptic phenotype of EAAT3-deficient mice. The role of EAAT3 in glutathione synthesis and protection from oxidative stress represents another activity that likely impacts the pathogenesis of epilepsy. Oxidative stress is a major component of epilepsy-driven pathology and represents a central process underlying the damaging effects of epileptic activity in the brain [240]. Increased oxidative stress is observed in virtually all epilepsy models [241] and in epileptic human brains [242]. Thus, protection from oxidative damage provided by EAAT3-mediated uptake of cysteine may be crucial to prevent neuronal cell death in epilepsy. In support of this hypothesis, mis-sorting of EAAT3 in SorCS2-deficient neurons caused enhanced oxidative stress and neuronal cell loss, as well as increased mortality in the murine PTZ-kindling model of epilepsy [144].

### 7.2. Alzheimer’s Disease

Alzheimer’s disease (AD) is an age-related dementia characterized by progressive formation of intracellular neurofibrillary tangles, extracellular amyloid β (Aβ) plaques, as well as synaptic dysfunction and neuronal cell loss. Several lines of evidence link AD pathology to neuronal hyperactivity and alterations in glutamatergic signalling [243]. Emerging evidence shows impaired glutamate uptake and altered glutamate-glutamine cycle in AD [244].

Defects in glutamate uptake as pathological mechanism in AD were supported by initial observations documenting reduced binding and uptake of aspartate, the second amino acid transported by EAATs in brain tissue from AD patients [155,245]. Additional studies showed impaired glutamate uptake into cultured astrocytes derived from AD patients [246]. Subsequent experiments documented decreased expression levels of glial glutamate transporters EAAT1 and EAAT2 in AD brains [177,178], although not all reports confirmed these findings [247]. More detailed analyses revealed that the astrocytic expression of EAAT2 was lower in AD subjects with dementia as compared to AD patients with no dementia [179], suggesting a protective role for EAAT2 expression in astrocytes in the AD brain.

Studies in mouse models of AD also in general point to reduced levels of glial EAATs as being associated with the disease. In a model with AD-associated mutant proteins overexpressed specifically in the hippocampi of adult mice, EAAT2 levels were lower [180]. Similar results were obtained in classical transgenic AD models with global overexpression of mutant proteins, in which a decrease in EAAT1 and EAAT2 levels was observed in hippocampi [181,182], along with reduced glutamate re-uptake activity [183] and altered glutamatergic neurotransmission [181].

It seems that Aβ, the causative agent in AD, might directly impact the expression and/or localization of EAATs, providing a molecular explanation for impaired glutamate re-uptake seen in AD. In cultured astrocytes, Aβ reduced EAAT1 and EAAT2 levels in some studies [182,184]. However, in another study Aβ had no impact on total levels of EAAT1 or EAAT2 but decreased the amount of EAAT2 at the cell surface of cultured astrocytes [185].

Functional studies in mouse models support the idea that EAAT2 activity protects the brain against AD-related dysfunctions. For example, loss of one allele of EAAT2 gene accelerated the onset of cognitive deficit in AD mice [186]. Furthermore, treatment with ceftriaxone to upregulate EAAT2 levels rescued cognitive decline in various transgenic mouse models of AD [182,187].

Apart from EAAT2, little is known about other EAATs in the context of AD pathology. EAAT3 aberrantly accumulates in hippocampal CA2-CA3 pyramidal neurons of AD patients [248]. Moreover, a Triton X-100-insoluble form of the transporter is significantly increased in the hippocampi of AD patients compared to controls but the relevance of this observation for EAAT3 function and AD pathogenesis remains unclear [248]. Also, the results of animal studies are conflicting. Schallier and colleagues reported an increase in EAAT3 expression in the hippocampi of AD mice [183], whereas a decrease in the hippocampal expression of EAAT3 in another AD model had been reported by Cassano et al. [181].

### 7.3. Parkinson’s Disease

Parkinson’s disease (PD) is a chronic degenerative disorder of the CNS that mainly affects the motor system and leads to a loss of dopaminergic neurons in the substantia nigra [249]. The aetiology of PD involves both genetic and environmental factors and is a result, at least partially, of oxidative damage to dopaminergic neurons [250]. Classical animal models of PD employ toxic insult to the substantia nigra by application of 1-methyl-4-phenyl-1,2,3,6-tetrahydropyridine (MPTP) or 6-hydroxydopamine (6-OHDA) [251].

As PD is associated with increased oxidative stress [250], a protective role for EAAT3 in the course of the disease seems plausible. In support of this hypothesis, acute induction of EAATs dysfunction in rats by injecting glutamate transporters inhibitor l-trans-pyrrolidine-2,4-dicarboxylate causes progressive loss of dopaminergic neurons, accompanied by GSH depletion, enhanced oxidative stress and excitotoxicity [252]. Potential contributions of individual EAATs to neuroprotection in PD have not been dissected thus far but, based on its physiological functions, this role can likely be attributed to EAAT3. In line with this concept, EAAT3 is expressed in dopaminergic neurons [188] and the knockout of *Eaat3* in mice causes an age-dependent loss of dopaminergic neurons in the substantia nigra that coincides with increased oxidative stress and inflammation [79]. These defects can be rescued by administration of N-acetylcysteine (NAC), a membrane-permeable cysteine precursor that by-passes the need for active uptake of cysteine into neurons [79]. NAC treatment also efficiently improves performance in a motor coordination test in MPTP mouse model that is characterized by decreased glutathione levels and increased oxidative stress in the midbrain region [188]. Interestingly, MPTP induces a shift of EAAT3 to the plasma membrane fraction, again pointing to EAAT3 sorting as a regulatory process during oxidative stress [188].

There is also some evidence for a protective role of EAAT2 in PD. In the 6-OHDA rat model, glutamate re-uptake was lower in the lesioned striatum [253]. Pre-treatment with ceftriaxone to increase EAAT2 levels and to enhance glutamate uptake prevented loss of tyrosine hydroxylase levels associated with 6-OHDA lesions [253].

### 7.4. Huntington’s Disease

Huntington’s disease (HD) is a neurodegenerative disorder associated with an aberrant expansion of glutamine repeats in the amino terminal domain of the huntingtin protein. Mutated huntingtin is toxic and causes loss of medium spiny neurons in the striatum [254]. Typically, mouse models of HD overexpress either exon 1 of mutant human huntingtin (R6/1 and R6/2 mice [255]) or the full-length human mutant huntingtin (YAC128 model [256]). Observations in in vitro studies, in transgenic models and in HD patients revealed alterations in glutamate transporter EAAT2 levels as being associated with this disease. Lower levels of the EAAT2 transcript were detected in HD patient tissues as compared to control brains [189]. Also, glutamate uptake into proteoliposomes derived from human brain tissue was reduced in HD as compared to control brains [257]. Furthermore, progressive loss of EAAT2 mRNA and protein was seen in cortex and striatum of the R6 mouse model, along with a reduction in glutamate transport capacity [190,191,192,193,196]. Of note, selective expression of mutant huntingtin in astrocytes was sufficient to reduce levels of EAAT2 protein and transcript and to cause HD phenotype in mice [194]. Conceivably, mutant huntingtin may directly reduce the expression of astrocytic glutamate transporters, resulting in loss of protective properties of glia in R6/2 mice [192]. This effect is presumably due to the reduced binding of the transcription factor Sp1 to the EAAT2 gene promoter resulting from sequestration of Sp1 by mutant huntingtin [194]. In YAC128 mice, no changes in EAAT2 levels were detected, although glutamate uptake in the brain was impaired. This finding was explained by decreased palmitoylation of EAAT2, a post-translational modification important for transporter function [258]. Overall, decreased glutamate uptake in glia caused by mutant huntingtin may critically contribute to excitotoxicity in HD. Along these lines, ceftriaxone treatment applied to increase EAAT2 levels restored glutamate uptake in the striatum and attenuated behavioural phenotype in HD mice [195]. On the other hand, loss of one *Eaat2* allele did not influence behavioural phenotypes of R6/2 mice [193]. Thus, the causative role of EAAT2 in the pathogenesis of HD still requires further elucidation.

Although expression studies gave conflicting results, showing either no change [191] or a decrease in EAAT3 levels [193] in cortex and striatum of HD mouse models, there is evidence for a role of this transporter in the pathogenesis of HD. Firstly, R6/1 mice show increased oxidative damage [196]. This oxidative damage, as well as HD-related behavioural phenotypes, can be ameliorated by treatment with NAC, suggesting failure of cysteine import into neurons as the underlying cause of neurodegeneration [196]. Moreover, neurons expressing mutant huntingtin show reduced amounts of EAAT3 at the cell surface. These alterations coincide with decreased cysteine uptake, lower glutathione levels and a higher degree of oxidative stress [145]. In detail, mutant huntingtin appears to alter intracellular trafficking of EAAT3 through Rab11+ recycling endosome, thereby limiting its activity at the plasma membrane and impairing neuronal cysteine import [145]. Thus, EAAT3 may have a protective role in HD, as it has in other neurodegenerative diseases associated with oxidative stress.

### 7.5. Multiple Sclerosis

Multiple sclerosis (MS) is a demyelinating disease with progressive neurodegeneration caused by an autoimmune response to self-antigens [259]. Pathological changes affect the brain, the spinal cord and the optic nerve. The underlying cause remains elusive but a crucial component of disease pathogenesis is neuroinflammation. The most frequently used animal model of MS is experimental autoimmune encephalomyelitis (EAE) which employs induction of myelin-targeting autoimmunity and results in CNS inflammation [260]. Since astrocytes are important players in the neuroinflammatory response in MS [261,262], astroglial EAATs have been studied for their involvement in the pathogenesis of this disease. Moreover, high levels of extracellular glutamate observed in diseased tissue presumably contribute to axonal damage, neuronal cell death and tissue degeneration in MS [263,264]. Thus, the glutamate re-uptake system is likely to play a protective role in MS.

Indeed, functional studies point to a link between glutamate uptake and the pathogenesis of MS. However, the direction of changes observed in animal models and in patients is counterintuitive as glutamate uptake activity is generally higher in MS tissues. Potentially, the primary alteration occurring in MS is elevation in extracellular glutamate concentrations that cannot be evened out by enhanced uptake. Glutamate uptake into the synaptosomes derived from the spinal cord is significantly higher in EAE than in control rats [197]. The same effect is observed for glutamate uptake into synaptosomes and glial plasmalemmal vesicles derived from EAE rat brains [198], as well as glial plasmalemmal vesicles from MS patients’ optic nerves [111].

Conceptually, enhanced glutamate uptake in MS tissue disagrees with the overall decreased total levels of EAAT1 and EAAT2 in MS models. In EAE, mRNA levels for EAAT1 increase but protein levels for EAAT1 and EAAT2 decrease [197]. Similarly, transcriptional upregulation of genes for EAAT1 and EAAT2 is seen in the forebrain and cerebellum of rat models [198,199] but protein levels of EAAT2 in cerebellum and of EAAT1 in cerebellum and forebrain are significantly reduced just before the acute phase of the EAE and during the recovery phase [199]. Intriguingly, expression levels of EAAT1 and EAAT2 (both transcript and protein) were higher in optic nerves isolated from MS patients as compared to control tissue [111]. In this study, EAAT1 expression was observed in oligodendrocytes, while EAAT2 was expressed in astrocytes in the human optic nerve [111].

Discrepancies between EAATs transcript and protein levels and glutamate transport activities suggest that expression levels of EAATs are highly dependent on the stage of MS and on the affected tissue and cell type analysed. Also, total levels of transporter transcript and/or protein might not accurately reflect the complex changes in transporter activity occurring in the diseased tissues. For example, in patient brain samples, EAAT2 immunoreactivity is decreased in MS lesions but enhanced in surrounding activated astrocytes [200], suggesting an active role of astrocytic EAAT2 in reaction to MS-related brain damage. An alternative explanation for the conflicting results regarding EAAT2 levels and glutamate uptake may be that other transporters—for example, EAAT3—are responsible for the increased glutamate uptake in MS tissues. Depending on the study, mRNA levels of EAAT3 were unchanged in optic nerves of MS patients [111], slightly upregulated in the brain of a rat EAE model [198] or dramatically increased in the spinal cord of EAE rats [197].

The protective role played by EAAT3 in oxidative stress suggests an alternative mode of action for this transporter in MS. Oxidative stress is an important component of MS pathology [265] and pharmacological treatment to increase levels of Nrf2 is protective in EAE [266]. As activation of the transcription factor Nrf2 results in induction of EAAT3 gene expression, it is tempting to speculate that this mechanism contributes to protecting axons from oxidative damage in EAE.

### 7.6. Amyotrophic Lateral Sclerosis

Amyotrophic lateral sclerosis (ALS) is a progressive nervous system disorder that causes degeneration of motor neurons in the brain and spinal cord and leads to loss of muscle control. Multiple gene mutations have been associated with this disease, with mutations in the gene encoding superoxide dismutase 1 (SOD1) being the first reported to cause ALS and contributing to 15–20% of the familial ALS cases [267]. However, in the majority of cases the aetiology of the disease remains less clear. It is believed that both genetic and non-genetic contributors play a causal role in ALS pathogenesis. Regardless of the aetiology, different types of ALS share features of oxidative stress, inflammation, glia activation and glutamate-driven excitotoxicity [268,269].

Excitotoxicity constitutes an important component of ALS pathology and multiple studies assessed glutamate levels in blood plasma and cerebrospinal fluid (CSF) of ALS patients and animal models. Plasma glutamate levels [270], as well as glutamate levels in the CSF [271] were substantially elevated in ALS patients when compared to controls, although these observations were not confirmed in all reports [272]. Moreover, high glutamate concentrations in the CSF correlated with a spinal onset of the disease, with more severe impairment of limb function and with a higher rate of muscle deterioration [273]. Plasma levels of glutamate also correlated with spinal onset of the disease [274]. Increases in glutamate levels were attributed to impaired glutamate uptake. In support of this assumption, a marked decrease in the maximal velocity of the transport for high-affinity glutamate uptake in synaptosomes from spinal cord, motor cortex and somatosensory cortex was observed in patients with ALS [275].

In line with impaired glutamate uptake in ALS, multiple studies reported decreased levels of EAAT2 in patients, in particular in the spinal cord. Thus, EAAT2 immunoreactivity was severely decreased in ALS, both in motor cortex and in spinal cord. By contrast, there was no change in EAAT1 levels and only a modest loss of EAAT3 in the motor cortex in ALS. This minor loss of EAAT3 may be attributed to loss of cortical motor neurons [201]. Later study confirmed the dramatic reduction of EAAT2 levels in the spinal cord of ALS patients [202]. More importantly, a mutation in the EAAT2 gene was identified in an ALS patient [203]. Subsequent studies showed that this mutation impairs glutamate transport likely due to defect in the glycosylation that reduces cell surface exposure of EAAT2 [204]. In addition, defects in splicing of EAAT2 transcripts seem to contribute to ALS pathogenesis. Thus, products of abnormal splicing of EAAT2 transcripts are seen in motor cortex, spinal cord and in CSF in a large fraction of ALS patients [205]. The presence of these aberrant transcripts correlates with loss of EAAT2 protein, as polypeptides produced from these transcripts are unstable and mis-localize to intracellular compartment [205].

Loss of EAAT2 was also noted in animal models of ALS. For example, decreased protein levels were observed in the dorsal spinal cord of SOD1 mutant mice [206] and rats [207]. More detailed analyses revealed that EAAT2 loss in the ventral horn of the spinal cord coincided with gliosis but preceded motor neuron degeneration. At early time points in the disease, EAAT2 was lost in a patchy pattern around motor neurons and the extent of loss and gliosis further progressed in the course of the disease [207]. Interestingly, down-regulation of EAAT2 expression was observed in the spinal cord but not in the motor cortex of SOD1 mutant mice, suggesting spatial specificity of the changes in EAAT2 expression levels [208,209].

What may be the primary cause of EAAT2 deficiency in ALS? Since no astroglia loss is observed in ALS, the dramatic decrease in EAAT2 levels likely reflects a primary event in the disease pathogenesis rather than a secondary consequence of neurodegeneration. Several cell-autonomous mechanisms underlying the loss of EAAT2 in SOD1 mutants were proposed. In Madin-Darby Canine Kidney (MDCK) cell line mutant SOD1 caused a reduction in EAAT2 levels, presumably by inducing internalization and lysosomal degradation of the transporter [210]. Similarly, overexpression of SOD1 mutants in transporter-expressing oocytes caused oxidation of EAAT2 and impaired glutamate uptake activity [211]. EAAT2 levels and glutamate uptake seem to be also directly influenced by another ALS-associated mutant protein TDP-43, an RNA binding protein involved in RNA processing. Astrocyte-specific expression of a disease-associated mutant TDP-43 resulted in progressive loss of EAAT1 and EAAT2 in the spinal cord of transgenic rats [212] and was sufficient to evoke neuronal cell loss and ALS symptoms in these animals [212]. As TDP-43-dependent processing of transcripts might play a role in EAAT2 biogenesis [276], mutant TDP-43 may well impair EAAT2 expression and contribute to the excitotoxicity seen in ALS.

The observation that the loss of EAAT2 from astrocytes causes symptoms of ALS argues for a causal role of EAAT2 depletion in the ALS pathogenesis. Indeed, loss of one *Eaat2* allele significantly accelerates the onset of neuronal cell loss in the spinal cord of ALS mice [209] whereas ceftriaxone-induced increase in EAAT2 levels is protective [83]. However, not all studies support the idea that increasing EAAT2 levels and/or activity may be a promising therapeutic strategy in ALS. Thus, overexpression of EAAT2 in SOD1 mutant mice was only partially protective [213]. Although increasing expression of EAAT2 prevented glutamate-induced toxicity in primary cortical cultures, it had only mild effects on motor performance and only partially protected motor neurons from neurodegeneration in SOD1 mutant mice in vivo. Moreover, increasing EAAT2 expression did not impact the onset of paralysis or decline in body weight or life span [213]. Also, focal restoration of EAAT2 expression in the spinal cord of ALS mice did not have any protective effects, although it increased the glutamate uptake capacity [214]. Finally, the results of a clinical study with ceftriaxone in ALS patients were disappointing. Despite promising stage-2 data, the stage-3 study failed to show clinical efficacy of ceftriaxone in ALS [215].

### 7.7. Cerebellar Diseases

Some diseases caused by dysfunctions of the cerebellum have been linked to EAATs, mainly to EAAT1 and EAAT4, which are highly abundant in this brain structure. The cerebellum plays a crucial role in movement coordination and its malfunction causes problems with voluntary movements. Impaired functions of the cerebellum often stem from altered activity and/or loss of Purkinje cells (PCs), the main type of neurons in the cerebellar cortex. Cerebellar diseases genetically or functionally linked to EAATs include Niemann-Pick disease type C and essential tremor, as well as various cerebellar ataxias which are degenerative disorders characterized by the presence of uncoordinated movements. Multiple types of cerebellar ataxias exist that result from a range of conditions and genetic defects. EAATs dysfunctions have been implicated in some of them, namely in subtypes of spinocerebellar ataxias and episodic ataxia type 6.

Episodic ataxia is a heterogeneous group of disorders of various origins. One of the subtypes—episodic ataxia type 6 (EA6)—is causally linked to mutations in the gene encoding EAAT1. Causal involvement of mutant EAAT1 in this disease was discovered in 2005, when a P290R mutation in the EAAT1 gene was found in a patient with episodic ataxia [216]. This mutation caused decreased expression of an EAAT1 variant with markedly reduced capacity for glutamate uptake [216]. Another mutation (C186S) in a highly conserved transmembrane region was later found in other cases of episodic ataxia characterized by milder phenotypes. The latter mutation also impairs glutamate uptake activity when expressed in COS7 cells [217]. Since EAATs also have a chloride channel activity, later studies aimed at verifying whether this function is also disrupted by the EA6-associated P290R mutation [218]. Indeed, in addition to reducing cell surface levels of EAAT1 and impairing glutamate uptake, the mutation modified the anion conductance and glutamate dependence of EAAT1 anion currents [218]. These data pointed to a complex mechanism underlying EA6 pathogenesis that involves both impaired glutamate uptake and alterations in anion conductance mediated by EAAT1. In line with studies in mammalian cell models, expression of the P290R variant of EAAT1 in glial cells of Drosophila larvae caused episodic paralysis and abnormal glia morphology. Presumably, this phenotype was not the result of impaired glutamate uptake but of abnormal chloride flow from CNS glia [219]. More recently, two new mutations were discovered in ataxic patients, encoding mutant V393I [220] and R454Q [221] variants of EAAT1. The functional implications of these mutations remain unknown so far.

Spinocerebellar ataxias (SCAs) is a heterogeneous group of inherited autosomal dominant disorders that constitute a major cause of cerebellar ataxia. Although SCAs are associated with mutations in various genomic loci, they share common pathogenic pathways [277]. Impaired expression and/or function of EAATs, in particular EAAT4, emerges as one of the important factors contributing to PC-degeneration in SCAs.

Several lines of evidence imply a role for EAAT4 in the pathogenesis of spinocerebellar ataxia type 5 (SCA5). SCA5 is caused by mutations in a gene encoding scaffold protein β-III spectrin [147,278]. The symptoms, including PCs loss and ataxia, can be recapitulated in mice by depleting β-III spectrin [224]. Evidence linking β-III spectrin loss with EAAT4 dysfunction includes studies in β-III spectrin knockout mice that show progressive impairment of glutamate uptake in cerebellar homogenates [224]. β-III spectrin stabilizes EAAT4 at the plasma membrane and thereby promotes its activity [146]. Consequently, the L253P mutant of β-III spectrin prevents correct targeting of EAAT4 to the plasma membrane [148]. In line with these findings, already in three-weeks-old β-III spectrin knockout mice, prominent loss of EAAT4 is visible along with a reduced spontaneous activity of Purkinje cells [224]. In a second mouse model with disrupted β-III spectrin expression, similar, although less dramatic, phenotypes were noted, with loss of EAAT4 visible in the cerebella of 1 year-old animals [225]. Marked differences in EAAT4 protein levels were also found in SCA5 autopsy tissues [147].

Another type of spinocerebellar ataxia—SCA1—was also linked to decreased EAAT4 levels. Mice expressing the ataxin-1 protein with pathological polyglutamine repeats specifically in PCs are the primary SCA1 model. They develop ataxia and loss of motor coordination characteristic of the human disease [279,280]. In the cerebella of mutant mice, a dramatic decrease in EAAT4 protein levels was noted [222]. An unbiased study to identify genes with changed expression levels in the SCA1 mouse model confirmed decreased EAAT4 levels in the diseased cerebellum [223]. In summary, since inactivation of EAAT4 results in PCs death in mice [68], it is plausible that EAAT4 loss contributes to disease progression in SCA1 and SCA5.

Several SCAs have also been linked to altered EAAT1 levels, and EAAT1 activity in Bergmann glia appears to play a protective role in cerebellar neurodegeneration. Thus, SCA5 models are characterized by relatively late and mild loss of EAAT1, visible at 12 weeks or 1 year in β-III spectrin depleted mice, depending on the model [224,225]. It is not entirely clear whether this late-onset decrease of glial EAAT1 has any relevance for the disease progression but knocking out the EAAT1 gene accelerates PCs loss in young β-III spectrin knockout animals, specifically in the posterior cerebellum [226]. Also in SCA1 models, loss of Purkinje neurons correlates with decreased EAAT1 expression in Bergmann glia [227]. In SCA7 mouse models expressing mutant polyglutamine-expanded ataxin-7 in Bergmann glia, reduced glutamate uptake into cultured Bergmann glia and decreased levels of EAAT1 in the cerebellum are observed [228]. These defects coincide with cerebellar PCs loss and ataxia typical of this disease model. Thus, cerebellar EAAT1 may contribute to the protective role of Bergmann glia in prevention of neurodegeneration. The concept that EAAT1 plays a protective role in the cerebellum is further supported by observation from Fragile X-associated tremor/ataxia syndrome (FXTAS) patients. FXTAS cerebella are characterized by severe degeneration coinciding with loss of EAAT1 expression [281].

Essential tremor (ET) is a neurological disorder that causes involuntary and rhythmic shaking. ET is one of the most common neurological disorders and its prevalence is increasing with age [282]. A growing body of evidence now links ET with a pathological alteration in cortico-subcortical communication but also with abnormal functions of the cerebellum [283]. One postulated mechanism for ET pathogenesis is excitotoxic death of PCs, which could be caused by impaired glutamate uptake. Polymorphisms in the gene encoding EAAT2 had been associated with ET [284] but subsequent studies gave inconsistent results [285,286,287]. Meta-analysis of various genetic association studies now suggests that this polymorphism in the EAAT2 gene locus is not associated with the risk for ET [288]. Still, EAAT2 levels are decreased in ET cerebella compared to control tissues, while EAAT1 protein levels remain unchanged [289,290]. The pathophysiological relevance of these findings need to be clarified.

Niemann-Pick disease type C (NPC) is a rare autosomal recessive lysosomal storage disorder. It is characterized by a range of clinical features, including severe progressive neurodegeneration and ataxia due to PCs loss [291]. The majority of cases are caused by mutations in the gene encoding NPC1. Mouse models of the disease are either genetically deficient for wildtype *Npc1* or express the D1005G NPC1 mutant protein (*Npc1*(nmf164) strain) [292]. *Npc1*(nmf164) mice show defects in Bergmann glia morphogenesis resulting in decreased levels of EAAT1 in the cerebellum [292]. In line with these findings, levels of EAAT1, EAAT2 and EAAT4 are decreased in the cerebella of NPC1-KO mice, accompanied by loss of β-III spectrin and by chloride-dependent alterations in PCs activity patterns [293]. These alterations mirror the molecular phenotype of SCA5, suggesting that NPC and SCA5 share pathogenic pathways.

### 7.8. Ischemic Stroke

Ischemic stroke is characterized by the sudden disruption of blood circulation in an area of the brain, resulting in loss of oxygen and glucose supply. As a consequence, cellular homeostasis is altered inducing a cascade of pathological events that cause neuronal cell loss [294]. Among the various factors contributing to brain damage in ischemic stroke [295], glutamate excitotoxicity and oxidative stress provide direct molecular links to EAATs functions.

Mainly glial EAAT2 and neuronal EAAT3 have been studied in the context of ischemic stroke. Interestingly, a polymorphism in EAAT2 gene promoter was associated with increased plasma glutamate concentrations and with a higher frequency of early neurological worsening in human stroke patients [229]. Moreover, EAAT2 levels decreased in the hippocampus within a few hours after ischemia in rats while EAAT1 and EAAT3 remained unchanged [230]. Decreasing EAAT2 levels even further by antisense RNA-mediated knockdown exacerbated neuronal damage in the rat brain induced by transient focal cerebral ischemia and increased mortality rates [231]. Conversely, ceftriaxone pre-treatment of rats prevented the post-stroke decrease in EAAT2 levels, ameliorated neurological deficit 24 h after ischemia and decreased infarct volume [232]. The peroxisome proliferator-activated receptors γ (PPARγ) agonist rosiglitazone also induced expression of EAAT2, promoted glutamate uptake and was protective in an in vitro model of ischemic stress (OGD) in neuronal-astrocyte co-cultures [233]. At present, it cannot be excluded that other PPARγ targets induced by rosiglitazone treatment also contribute to neuroprotection. Still, EAAT2 decrease appears to be detrimental and EAAT2 may well play a protective role after stroke, if its expression could be sustained. A protective role for EAAT2 after ischemic stroke presumably results from its ability to clear up toxic glutamate from the extracellular space.

A role for EAAT3 in post-stroke recovery seems even more likely because of its function in protection from oxidative stress. EAAT3 expression is induced following ischemia and studies in knockout mice lacking EAAT3 support a protective role of this transporter under ischemic conditions. For example, Won and colleagues demonstrated that EAAT3-KO mice subjected to transient cerebral ischemia exhibit much more widespread hippocampal neuronal death than wild-type mice, along with more oxidative stress and reduced neuronal glutathione levels [234]. Treatment with the cell-permeable cysteine precursor NAC restored neuronal glutathione levels and prevented neuronal death, supporting the hypothesis that impaired cysteine uptake in EAAT3-depleted neurons underlies the higher susceptibility to oxidative damage after stroke. Also, EAAT3-KO mice show slightly larger infarct volumes and more severe neurological deficit 24 h post focal brain ischemia and 4 weeks after stroke, as compared with controls [235]. Another study showed poor survival of newborn neurons generated in the hippocampus after ischemia in EAAT3-deficient mice [236]. Still, some studies failed to confirm the relevance of EAAT3 activity for neuronal survival after stroke. For example, antisense knockdown of EAAT3 did not impact transient focal cerebral ischemia-induced neuronal damage in rat brain [231]. Thus, more studies are required to substantiate a possible involvement of EAAT3 in ischemic stroke.

## 8. Summary

Although originally discovered as transporters for excitatory amino acids, EAATs show interesting additional properties with high relevance for human (patho)physiology. Chloride channel activity emerges as a main physiological activity of EAAT4 and EAAT5. Import of cysteine into neurons to ensure glutathione synthesis for oxidative stress response appears to be a crucial role for EAAT3. Accordingly, the mechanisms of EAATs involvement in pathogenesis of CNS diseases are manifold but also offer exciting perspectives for therapeutic intervention that warrant further exploration.

Foremost, EAAT2 already receives a lot of attention as a potential target in treatment of CNS pathologies [296]. Due to its ability to prevent toxic glutamate accumulation in the extracellular space, it plays a protective role in multiple disorders of the CNS such as Huntington’s disease, Alzheimer’s disease and amyotrophic lateral sclerosis. The availability of the FDA-approved drug ceftriaxone to induce its expression makes EAAT2 an attractive target for potential therapies, although its applicability to a range of neurodegenerative disorders associated with excitotoxicity still remains to be proven.

EAAT3 emerges as a promising therapeutic target for preventing oxidative damage to neurons; a process relevant for multiple brain pathologies, in particular neurodegenerative disorders, epilepsy and ischemic stroke. Pharmacological strategies to modulate its functional expression in the human brain are not in place as yet. Still, pathways inducing EAAT3 expression upon oxidative stress as well as the mechanisms of its intracellular sorting have been a subject of extensive studies that are likely to uncover effective means to modulate EAAT3 activity in patients.

## Figures and Tables

**Table 1:** Nomenclature and properties of EAATs.

Protein Name (Human)	Protein Name (Rodent)	Gene	Main Biological Activity	Predominant Expression Pattern in the Mature Brain	
				Brain Regions	Cell Type and Subcellular Localization
EAAT1	GLAST	SLC1A3	glutamate transporter	cerebellum	astrocytes (perisynaptic)
EAAT2	GLT-1	SLC1A2	glutamate transporter	whole brain	astrocytes (perisynaptic); axon terminals (presynaptic)
EAAT3	EAAC1	SLC1A1	glutamate and cysteine transporter	whole brain	neurons (postsynaptic, cell soma and dendrites)
EAAT4	EAAT4	SLC1A6	glutamate transporter; glutamate-gated chloride channel	cerebellum	neurons (postsynaptic, dendritic spines)
EAAT5	EAAT5	SLC1A7	glutamate-gated chloride channel	retina	neurons (presynaptic)

## Abbreviations

6-OHDA	6-hydroxydopamine
AD	Alzheimer’s disease
ALS	amyotrophic lateral sclerosis
AMPAR	α-amino-3-hydroxy-5-methyl-4-isoxazolepropionic acid receptor
ARE	antioxidant responsive element
CNS	central nervous system
CSF	cerebrospinal fluid
EA	episodic ataxia
EAE	experimental autoimmune encephalomyelitis
EAAT	excitatory amino acid transporter
ET	essential tremor
GABA	γ-aminobutyric acid
GLAST	glutamate-aspartate transporter
GLT-1	glutamate transporter 1
GSH	glutathione
HD	Huntington’s disease
KO	knockout
mGluR	metabotropic glutamate receptor
MPTP	1-methyl-4-phenyl-1,2,3,6-tetrahydropyridine
NAC	N-acetylcysteine
NMDAR	*N*-methyl-d-aspartate receptor
NPC	Niemann-Pick disease type C
Nrf2	nuclear factor (erythroid-derived 2)-like 2
OGD	oxygen-glucose deprivation
PC	Purkinje cell
PD	Parkinson’s disease
PKA	protein kinase A
PKC	protein kinase C
PMA	phorbol 12-myristate 13-acetate
PPARγ	peroxisome proliferator-activated receptors γ
PTZ	pentylenetetrazol
SCA	spinocerebellar ataxia
SE	status epilepticus
